# Distinct functional enrichment of transcriptional signatures in pigs with high and low IFN-gamma responses after vaccination with a porcine reproductive and respiratory syndrome virus (PRRSV)

**DOI:** 10.1186/s13567-016-0392-3

**Published:** 2016-10-20

**Authors:** Tahar Ait-Ali, Ivan Díaz, Ferran Soldevila, Esmeralda Cano, Yanli Li, Alison D. Wilson, Bruno Giotti, Alan L. Archibald, Enric Mateu, Laila Darwich

**Affiliations:** 1 The Roslin Institute and Royal (Dick) School of Veterinary Studies, University of Edinburgh, Midlothian, EH25 9RG UK; 2 Centre de Recerca en Sanitat Animal (CReSA)- IRTA, Campus de la Universitat Autònoma de Barcelona (UAB), 08193 Cerdanyola del Valles, Spain; 3 Virology Department, Animal and Plant Health and Agency, Addlestone, KT15 3NB UK; 4 Department of Pathology and Pathogen Biology, Royal Veterinary College, Hatfield, AL9 7TA UK; 5 Department Sanitat i Anatomia Animals, Faculty of Veterinary, UAB, 08193 Cerdanyola del Valles, Spain

## Abstract

Little is known about the host factor in the response to PRRSV vaccination. For this purpose, piglets were immunized with a commercial PRRSV-live vaccine and classified as high responders (HR) or low responders (LR) as regards to the frequencies of virus-specific IFN-γ-secreting cells. Six weeks post vaccination, PBMCs isolated from three individuals with the most extreme responses in each HR and LR groups and 3 unvaccinated controls, were either stimulated with phytohaemagglutinin, challenged with the vaccine or mock treated for 24 h, prior conducting transcriptional studies, gene ontology and pathway analyses. The LR group had very low neutralizing antibody levels and showed a higher number of down-regulated transcripts compared with the HR group (FDR < 0.2, *P* < 0.001). Down-regulated genes encoded chemoattractants, proinflammatory cytokines and the interferon-inducible GBP family, and showed enrichment in wounding (FDR < 3.6E-13), inflammation (FDR < 8E-12), defence (FDR < 8.7E-09) and immunity (FDR < 7.6E-08), suggesting immune response impairment. In the HR group, down-regulated genes were involved in protein transport (FDR < 4.77E-03), locomotory behavior (FDR < 5.47E-3), regulation of protein localization (FDR < 1.02E-02), and regulation of TNF superfamily member 15 and miR181. In contrast, the HR group presented up-regulated transcripts associated with wounding (FDR < 4.95). Moreover, IFN-γ was predicted to be an inhibited upstream regulator since IFN-γ pathways were associated with higher number of down-regulated genes in the LR (*n* = 40) than the HR (*n* = 10). Divergent responses to PRRSV-vaccination may be the result of the genetic background of the host.

**Electronic supplementary material:**

The online version of this article (doi:10.1186/s13567-016-0392-3) contains supplementary material, which is available to authorized users.

## Introduction

Porcine reproductive and respiratory syndrome (PRRS) is arguably the most endemic infectious disease challenge for the pig industry and causes very significant economic losses worldwide [1, 2]. The causative agent is an enveloped, single-stranded 15-kb positive-sense RNA virus belonging to the Arteriviridae family in the order Nidovirales [3] known as PRRS virus (PRRSV). The extensive genetic diversity characteristic of PRRSV encodes considerable antigenic diversity which presents a highly variable challenge to the host’s immune system [4–12]. Moreover PRRSV is known to modify the host’s innate immune response, particularly by inhibiting type-I interferons (IFNs), affecting cytokine production or by down regulating toll-like receptor (TLRs) expression [13–19].

The most widely used strategy for reducing the incidence and limiting the impact of PRRSV-infection damages is vaccination. Several commercial vaccines (including live attenuated and inactivated) are available on the market but their efficacies are considered to be at best partial as they cannot provide full protection against the wide diversity of PRRSV strains circulating in the field [20, 21]. PRRSV vaccines are routinely applied to breeding herds to prevent reproductive problems but the beneficial effect of vaccination to piglets is still controversial. After immunization of a group of pigs with a given vaccine, a variety of immune response are often generated among pigs and only some of them are being protected against infection. Thus, the genetics of the host (pigs) and of the pathogen (PRRSV) appear to play an important role on the efficacy of vaccination in the control of PRRS.

The interaction of PRRSV with the immune system is believed to be of critical importance for defining immunological and clinical outcomes of the infection and, along with other factors, is associated with an inefficient development of the adaptive immunity. The prototypical adaptive response to PRRSV is characterized by a weak and delayed production of neutralizing antibodies and by relatively low levels of IFN-γ producing T-cells [22, 23]. Moreover, there is increasing evidence that the genetic variation of both host and virus also have impacts on the response to infection [24–29].

Therefore, in order to understand immune responses to PRRSV infection and/or vaccination it is necessary to examine both sources of variation: host (pig) and pathogen (PRRSV). Analysis of the host response using transcriptional profiling of pigs with different immune status against PRRSV may help to understand why vaccines offer only partial protection. The objective of this study was to identify transcriptional signals and molecular pathways associated with responses to a PRRSV vaccine [30]. Systems biology approaches have been used successfully to identify early gene signatures that predict immune responses in humans vaccinated with the live attenuated vaccine YF-17D against yellow fever virus and inactivated seasonal influenza vaccine against influenza virus with accuracy [31, 32]. In order to explore this approach for PRRSV vaccines pigs were vaccinated with a modified live (ML) PRRSV vaccine and later classified into two groups, namely “high responders” (HR) and “low responders” (LR) on the basis of the frequencies of virus-specific interferon-γ secreting cells (IFN-γ SC). Subsequently, the humoral response against the virus and the transcriptional profiles of peripheral blood mononuclear cells (PBMC) of both groups were examined in order to identify genes, molecular functions and biological pathways associated with the vaccine response.

## Materials and methods

### Animals and study design

**Figure 1:**
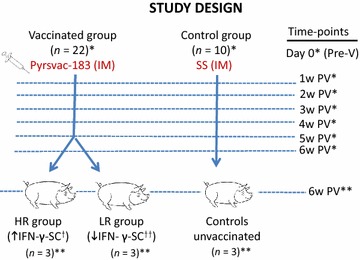
**Scheme of the study design.** Firstly, pigs were distributed in two groups: intramuscularly (IM) inoculated either with Pyrsvac-183 vaccine (*n* = 22) or sterile solution (SS) as unvaccinated control group (*n* = 10). Animals were followed-up from day 0 (pre-vaccination) to 6 weeks post-vaccination (PV). Blood and sera samples were collected every week for immunological determinations (ELISPOT virus-specific IFN-γ secreting cells -SC-, neutralizing antibodies -NA- assays, ELISA total Ab). At week 6 PV, vaccinated pigs with the highest and lowest average scores for the IFN-γ-SC were divided into the High Responder (HR) and the Low Responder (LR) groups, respectively. At this last time point, gene expression was analysed in PBMCs from the LR (*n* = 3), HR (*n* = 3) and control unvaccinated (*n* = 3) pigs using Affymetrix microarray platform. *Immunological studies: cellular and humoral immune responses (serum, PBMCs). **Transcriptional studies (microarray analysis using the PBMCs) HR: high responders; LR: low responders; ^†^The highest and the ^††^lowest average levels of virus-specific IFN-γ secreting cells through the time course of the study. Pre-vaccination (Pre-V). Post-vaccination (PV). Intramuscular (IM). Sterile Saline solution (SS).

Twenty-two healthy four-week-old Landrace × Large White PRRSV-negative pigs (as determined by serology and RT-PCR) were vaccinated intramuscularly with a genotype-1 commercial ML vaccine (Pyrsvac-183) according to the manufacturer’s instructions. Pigs were followed up for 42 days after vaccination. In parallel, 10 control pigs were subjected to a mock vaccination with sterile saline solution (SS). Pigs were bled for immunological determinations at day 0 (pre-vaccination) and then once a week post-vaccination (PV) for 6 weeks: days 7, 14, 21, 28, 35 and 42 PV (Figure 1).

### Peripheral blood mononuclear cell isolation

Heparinized blood samples were used for separation of PBMC by density-gradient centrifugation with Histopaque 1.077 (Sigma). PBMC were subsequently cultured using RPMI medium supplemented with 10% foetal calf serum (FCS) (Invitrogen, Madrid, Spain), 1 mM non-essential amino acids (Invitrogen), 1 mM sodium pyruvate (Invitrogen), 5 Mm 2-mercaptoethanol (Sigma), 50 000 IU penicillin (Invitrogen), 50 mg streptomycin (Invitrogen) and 50 mg gentamicin (Sigma) (complete RPMI). Trypan blue was used to assess cell viability.

### ELISPOT assays

ELISPOT assays were performed as described previously [12, 33] using commercial mAbs (P2G10 and biotin P2C11; BD Biosciences Pharmingen). Peripheral blood mononuclear cells (PBMC) were stimulated for 24 h (5 × 10^5^ PBMC/well, in triplicate) with the ML vaccine strain at a multiplicity of infection (m.o.i.) of 0.1 as a recall antigen, phytohaemagglutinin (PHA, 10 μg/mL) or culture medium. To calculate the frequencies of virus specific IFN-γ-SC, average counts of spots in unstimulated wells were subtracted from average counts obtained in antigen stimulated wells. Results were expressed as the number of PRRSV-specific IFN-γ-SC per 5 × 10^5^ PBMC.

**Figure 2:**
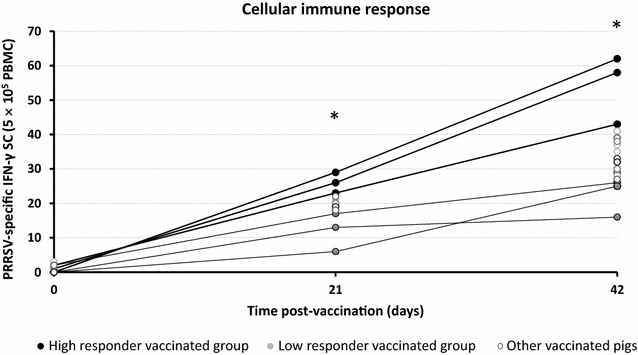
**Levels of IFN-γ secreting cells (SC) in all vaccinated pigs against the PRRSV vaccine strain at different time-points post-vaccination by ELISPOT.** Vaccinated pig groups were classified according to the intensity of the cellular mediated immune responses against the vaccine: “High responder” (HR) group comprised by pigs with the highest levels of IFN-γ-SC responses (black circle), the “Low responder” (LR) group with pigs with the Lowest IFN-γ-SC levels (grey circle) and other vaccinated pigs with intermediate IFN-γ-SC responses (white circle). The unvaccinated control group was not shown in the graph since all animals showed no specific response. *Statistical differences (*P* < 0.05).

Three pigs with the highest virus-specific IFN-γ-SC average responses throughout the timecourse of the study, designated as HR (High responders), and 3 pigs with the lowest average levels, or LR (Low responders), were selected for further analysis (Figure 1). Average HR and LR frequencies at 21 days PV were 26 ± 3/5 × 10^5^ cells versus 12 ± 5.6/5 × 10^5^ cells and at 42 days, 54.3 ± 8.1/5 × 10^5^ cells versus 22.3 ± 4.5/5 × 10^5^ cells (Figure 2). Three healthy unvaccinated pigs (SS) were randomly taken as a control group.

### Humoral response

A commercially available ELISA (Idexx PRRS X3 Ab Test) was used for measuring non neutralizing anti-PRRSV antibodies in blood samples taken at the different time-points of the study. Results were reported as a ratio of optical densities between the results of a given sample and the positive control (S/P ratio) included in the kit (cut-off: S/*P* ≥ 0.4). All samples were tested the same day and in the same batch of plates.

In parallel, homologous (against the vaccine virus) neutralizing antibodies (NA) titers were determined in a viral neutralization test (VNT). The VNT assays were done in MARC-145 cells using log_2_ serial dilutions (from 2^−1^ to 2^−8^) as described before running samples in duplicate [34, 35].

### In vitro treatment of peripheral blood mononuclear cells

At 6 weeks PV (Figure 1), PBMC isolated from the selected pigs of each group (HR and LR) and from unvaccinated pigs were seeded at 5 × 10^5^ cells/well in 96 well-plates and were cultured in triplicates with complete RPMI alone (negative controls), complete RPMI with phytohaemagglutinin (PHA, 10 μg/mL, positive controls) or the PRRSV vaccine strain (m.o.i. 0.1, specific stimulus) for 24 h. After that period, cells were harvested, centrifuged and cell pellets were resuspended in 1 mL of Trizol (Life Technologies) and frozen at −80 °C until processing for RNA extraction, microarray and downstream analysis.

### RNA extraction

Total RNA from PBMC of selected group pigs at 6 weeks PV was extracted using Trizol (Invitrogen, Paisley UK) according to the manufacturer’s instructions and was later purified using the Qiagen RNeasy minikit (Qiagen, Crawley, UK). RNA was eluted from the spin column in 30 µL of RNase-free water and stored as aliquots at −80 °C. The quantity and quality of RNA were assessed using a Nanodrop spectrophotometer (NanoDrop Technologies Inc, Wilmington, DE, USA) and Agilent 2100 bioanalyser (Agilent Technologies, Palo Alto, CA, USA) respectively.

### Microarray platform and data analysis

The extracted RNA was then analysed using the Affymetrix Snowball GeneChip® [36]. This GeneChip comprises 23 937 probe sets that interrogate approximately 23 256 transcripts from 20 201 *Sus scrofa* genes. Sense-strand cDNA was generated from total RNA (500 ng) subjected to two rounds of amplification (Ambion® WT Expression Kit). The resulting cDNA was used for biotin labelling and fragmentation according to the Affymetrix GeneChip® WT Terminal Labelling and Hybridization protocol (Affymetrix UK, High Wycombe). Biotin-labelled fragments of cDNA (5.5 μg) were hybridized to Affymetrix Snowball arrays using the Affymetrix HybWashStain kit and following the manufacturer’s recommendations. After hybridization, the arrays were washed and stained using the Affymetrix Fluidics Station 450 and then scanned in an Affymetrix 7G scanner. Image generation and the resulting CEL files for analysis were produced in AGCC—Affymetrix GeneChip Command Console Software. Initial QCs were performed in Expression Console. All microarray data used in the analyses herein are available from the Array Express repository [37]. The Affymetrix.CEL files were imported into the Partek Genomics Suite software package version 6.13.0213 (Partek, St. Louis, USA) for data analysis. Transcriptional responses were normalised to those from unvaccinated pigs prior to running an ANOVA analysis of the data (Additional file 1). Up-regulated and down-regulated differentially expressed transcripts in HR and LR were selected for further consideration if the false discovery rate (FDR) was ≤0.2.

For network analysis, the normalised array data were uploaded to the software Biolayout Express3D as described previously [36, 38]. Expression variations across treated groups were generated using the gplots package in R.

### Quantitative real time RT-PCR validation (qRT-PCR)

The differential expression of several selected genes, as identified from the microarray data, was verified at various time points using qRT-PCR. Reverse transcription was performed as described previously [26, 27]. Briefly, one microgram of total RNA was reverse transcribed using a TaqMan kit (Applied Biosystems, Foster City, CA, USA). For qRT-PCR, Platinum SYBR Green PCR SuperMix UDG was used, as described above. The qRT-PCR was performed with a Stratagene MX3000P (Stratagene, La Jolla, CA, USA). In Table 1 is showed the information about primer sequences of the selected genes. Samples were tested in triplicate, GAPDH served as the housekeeping gene and results were calculated as described previously [26, 27].

**Table 1:** **List of the genes selected for the quantitative real time RT-PCR validation**

Gene name	Sequence of the primers	Ensembl ID	Primer position	Description
GBP2	F: CCTGTGGTGGTGGTGGTTAT	ENSSSCG00000006923	Exon 3–4	Guanylate binding protein 2, interferon-inducible
	R: AGATGCCCTTCGTGTGAGAC			
IL1A	F: CAAGGACAGTGTGGTGATGG	ENSSSCG00000008090	Exon 4–5	Interleukin 1, alpha
	R: GTTGCTGATCTGGGCTTGAT			
IL8	F: CCTTCTTGGCAGTTTTCCTG	ENSSSCG00000008953	Exon 1–2	Interleukin 8
	R: AATTTGGGGTGGAAAGGTGT			
CCL4	F: CATGAAGCTCTGCGTGACTG	ENSSSCT00000019264	Exon 1–2	Chemokine (C–C motif) ligand 4
	R: ACGGTGTATGTGAAGCAGCA			
SAA1	F: ACTATGATGCTGCCCAAAGG	ENSSSCT00000014601	Exon 1–2	Serum amyloid A1
	R: ACTCCGTGGCCACTGTCTC			
IGF1	F: AGTTCGTGTGCGGAGACAG	ENSSSCT00000000935	Exon 2–3	Insulin-like growth factor 1
	R: GCCTCCTCAGATCACAGCTC			
CCL2	F: CTTCTGCACCCAGGTCCTT	ENSSSCT00000019290	Exon 1–2	Chemokine (C–C motif) ligand 2
	R: TGCTGCTGGTGACTCTTCTG			
IL1B	F: AGTGGAGAAGCCGATGAAGA	ENSSSCT00000008861	Exon 4–5	Interleukin 1, beta
	R: CATTGCACGTTTCAAGGATG			
LTBP1	F: GGGAACACCACCACTCTCAT	ENSSSCT00000009313	Exon 1–2	Latent transforming growth factor beta binding protein 1
	R:TTGTCCCTTGAACTGCACTG			

### Gene ontology and pathway analysis

Gene ontology and pathway analysis were carried out using DAVID bioinformatics resources [39] and Ingenuity pathway analysis [40], respectively. In particular for the identification of upstream regulators using IPA, expression datasets for the HR and the LR pigs were analysed independently with Fisher’s exact test as described by the software. The activation z-score was used to infer the state of activation of upstream regulators based on a comparison with a model that assigns random regulations. The *P* value overlap, which indicates possible upstream regulators, represents the significance of the overlap between the dataset genes identified and the known targets of transcriptional regulators. Differences were considered significant with *P*-value <0.05 and activation Z-score ≥2.

### Statistical analysis

Comparison of frequencies of IFN-SC, S/P ratios or titres of NA between groups was done using StatsDirect3 (v3.0.97) and the Kruskal–Wallis test. The coefficient of correlation (R^2^) between microarray and qRT-PCR techniques was produced by plotting the relative level of gene expression as assessed by microarray against the transcript levels generated by qRT-PCR.

## Results

### Characterization of the humoral response in HR and LR pigs

**Figure 3:**
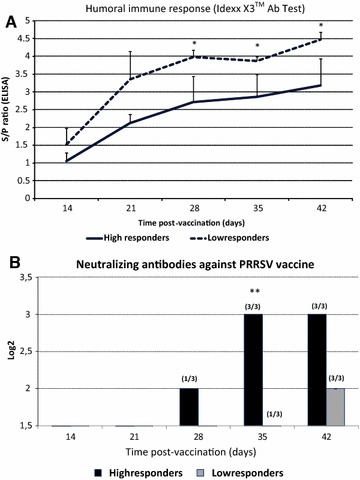
**Levels of humoral immune response in sera of High and Low responders after PRRSV vaccination. A** Total non-neutralizing anti-PRRSV antibodies by ELISA (Idexx PRRS X3 Ab Test). Cut-off: S/P ≥ 0.4. *Statistical differences by Kruskal–Wallis (*P* < 0.05). **B** Homologous Neutralizing Antibody (NA) titers in the viral neutralization test done in MARC-145 cells using log_2_ serial dilutions. Number of pigs presenting NA titers out of the total pigs is represented in brackets above the corresponding bar. *Statistical differences (*P* < 0.05). All samples from the control unvaccinated group and samples at day 0 post-vaccination of HR and LR groups were negative for all tests.

When the S/P ratios, corresponding to non-neutralizing antibodies (NA), were examined, values were always higher (*p* < 0.05) in the LR vaccinated group compared to the HR vaccinated group from day 14 PV onwards (Figure 3A). In contrast, HR had an earlier and stronger NA response to PRRSV vaccine at 28, 35 and 42 days PV in comparison to the LR group (Figure 3B). Regarding the viremia, CT means for HR were 28.3 ± 1.8 (day 14); 27.8 ± 1.7 (day 21); 29.9 ± 3.9 (day 28); and 34.0 ± 5.0 (day 35). For LR, CT means were 30.9 ± 3.4 (day 14); 28.4 ± 5.7 (day 21); 32.9 ± 1.8 (day 28); and 34.7 ± 1.6 (day 35). All animals were negative for PRRSV vaccine virus in blood at day 42, with the exception of one animal of the LR group that showed a CT = 35 that corresponds to 1.2–1.3 log10 genomic equivalences per mL.

### Host transcript regulation during PRRSV vaccine challenge

**Figure 4:**
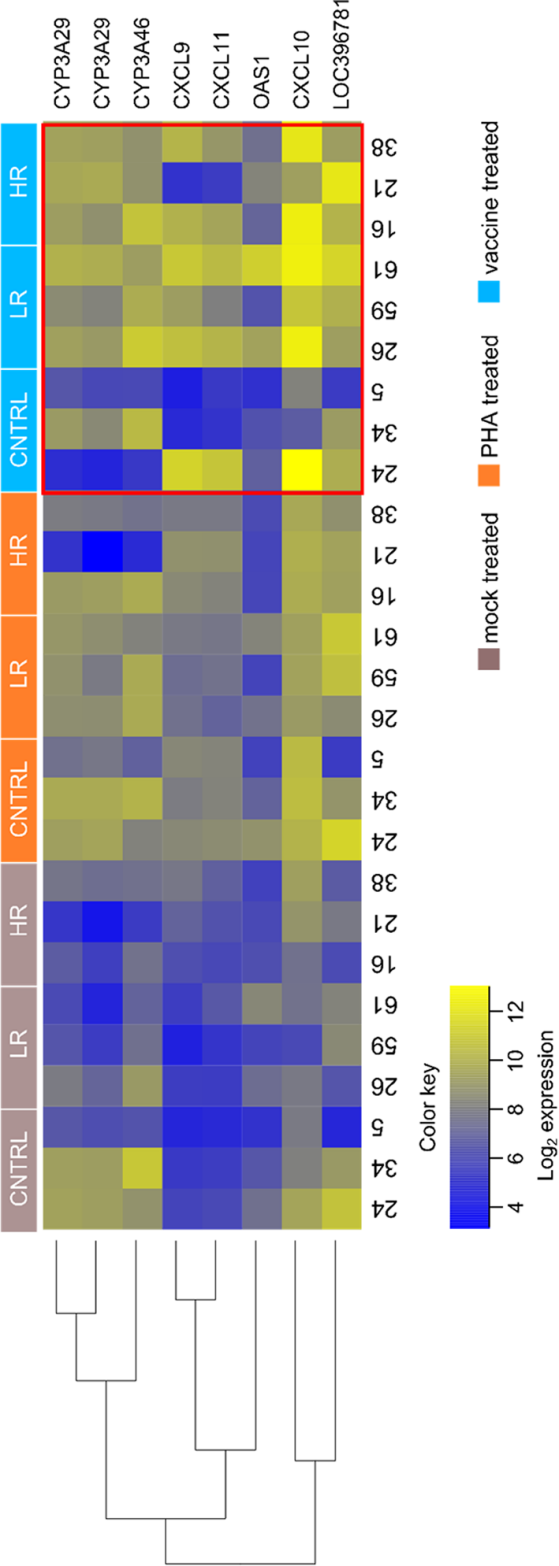
**Heatmap of probesets showing individual Log2 fold gene expression level with the different treatments and the pig groups** (unvaccinated control, CNTRL; Low Responder, LR; High Responder, HR). Pig identification is shown below heatmap: pig number 24, 34 and 5 for control pigs; pig number 26, 59 and 61 for LR pigs; pig number 16, 21, 38 for HR pigs. Rows, representing gene symbols for each probeset except for LOC396781, were ordered by hierarchical clustering and columns were displayed ordered by treatment (mock, in grey; PHA, in orange; vaccine, in blue). Log2 expression scale is shown. A red line box highlights heatmap of groups treated with vaccine.

From the total number of evaluated genes, 29 and 127 transcripts were identified as differentially expressed in the mock stimulated PBMC cultures of HR and LR vaccinated pigs, respectively (ANOVA FDR <0.2, *P* < 0.001). The proportion of genes which showed reduced levels of expression either in the presence of PHA treatment or in mock-stimulated cultures was larger in the LR vaccinated group compared with the HR (Table 2). In contrast, PBMCs from HR and LR vaccinated pigs and treated in vitro with the vaccine did not harbor statistically different regulated transcripts when compared to the controls (Table 2). To investigate further this observation, we examined differential expression using network analysis with Biolayout Express3D. Nine probesets mapping to 7 unique gene symbols showed a differential expression of >2 Log fold change between the PBMCs of LR and HR vaccinated pigs and unvaccinated control groups after vaccine stimulation (Figure 4, red squared section). Most of them showed increased expression in LR and HR groups compared to controls. Among these genes we found three chemokines, CXCL9, CXCL10 and CXCL11, which activate monocytes and T-lymphocytes, two Cytochromes, CYP3A46 and CYP3A29 of which the latter has been reported to be regulated by interferon [41], and OAS1 which is an interferon-induced antiviral enzyme [42].

**Table 2:** **Summary of the number of transcript regulated in PBMCs at 6** **weeks post-vaccination**

Group	Treatment	Comparator	Up-regulated transcripts (n)	Down-regulated transcripts (n)	Total genes (N)
High responder (HR)	Medium (mock)	Unvaccinated pigs	7	22	29
Low responder (LR) P	Medium (mock)	Unvaccinated pigs	18	109	127
High responder (HR)	PHA	Unvaccinated pigs + PHA	3	11	14
Low responder (LR) P	PHA	Unvaccinated pigs + PHA	4	20	24
High responder (HR)	PRRSV-vaccine	Unvaccinated pigs + vaccine	ns	ns	ns
Low responder (LR)	PRRSV-vaccine	Unvaccinated pigs + vaccine	ns	ns	ns

Non-redundant transcripts with ANOVA *P* value < 0.001 and a FDR < 0.2 were showed. Details of the genes for which changes in transcript levels were observed are presented in Additional file 1 which shows gene symbols, *P* values and fold changes.

ns: no statistically significant differences were detected between groups, HR: High responder group, LR: Low responder group.

FDR < 0.2, *P* < 0.001.

### Validation of differentially regulated transcripts using quantitative real time PCR (qRT-PCR)

Nine genes for which changes in transcript levels had been observed were selected for validation by qRT-PCR analysis. The selection criteria were: (i), differential regulation in the both groups (fold change ≥2.0, FDR ≤0.2) and ii), involvement in immune functions such as inflammation, antiviral responses and cell growth and recruitment molecules. Thus we selected the following genes: *GBP2*, *IL1A*, *IL8*, *CCL4*, *SAA1*, *IGF1*, *CCL2*, *IL1B* and *LTBP1*. The results showed positive correlation coefficients between the microarray and qRT-PCR techniques in the HR (R^2^ = 0.76) and LR (R^2^ = 0.86) groups (Additional file 2).

### Gene ontology analyses

**Figure 5:**
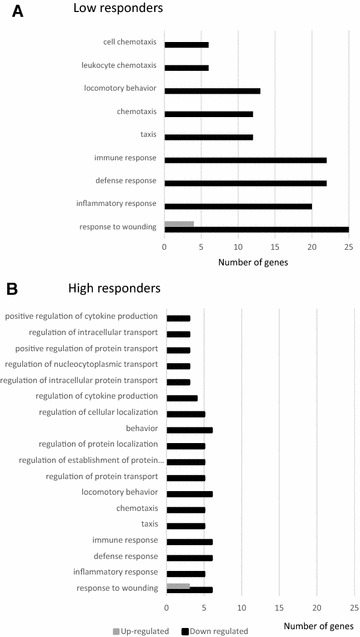
**Number of genes categorized by biological processes for each animal group.** Bars are representing the number of genes per category of biological process with a FDR ≤ 5% as shown in Additional file 3, for the Low responders (**A**) and the High responders (**B**) vaccinated groups.

Transcripts differentially regulated in the HR and LR vaccinated groups (Additional file 1) were analysed with respect to their biological processes, molecular functions and cellular (Additional file 3). Interestingly, the LR vaccinated group presented a higher number of down-regulated genes (6–25 genes) per biological processes category than the HR vaccinated (3–6 genes) but in a more restricted range of categories (9 categories vs 18 categories in the HR) (Figure 5). In the LR group mostly down-regulated transcripts showed significant fold functional enrichment (FFE) in 9 biological process involved in inflammation and immune responses as follow: response to wounding (8.6 FFE, FDR < 3.6E-13), inflammatory response (11.2 FFE, FDR < 8E-12), defence response (6.5 FFE, FDR < 8.7E-09) and immune response (5.4 FFE, FDR < 7.6E-08) (Additional file 3). Our results suggest that two broad molecular functions involved in chemokine receptor binding/activity and cytokine activity were down-regulated in LR and included genes encoding a wide range of chemoattractants (*CCL2*, *CCL17*, *CCL22* and *CCL23*) and the proinflammatory cytokines *IL1A* and *ILIB* and receptor antagonist *IL1RN*. Among the most down-regulated genes involved in immune response included several members of interferon-inducible guanylate binding protein (*GBP*) family *GBP2* (13.1 fold), *GBP4* (4.6 fold) and *GBP6* (9.9 fold). Finally, whereas no significant function dominated the category of up-regulated genes for the LR group, the greatest fold changes were observed for insulin-like growth factor 1 (*IGF1*, 13.0 fold).

In the HR vaccinated group, although the number of genes that were down-regulated per category was lower than the LR, the number of biological processes involved was twice as great as in the LR vaccinated pigs (Figure 5). The biological functional fold enrichments were associated with the regulation of protein transport, locomotory behaviour and regulation of protein localization which were enriched 42.4 FFE (FDR < 4.77E-03), 21.2 FFE (FDR < 5.47E-3) and 35 FFE (FDR < 1.02E-02), respectively. It is noteworthy that down-regulated genes in this group harboured the same trend than in the LR vaccinated group. For example SAA1, CCL3L1 and CCL4 were down-regulated group (Additional file 3). Also transcripts encoding for the tumor necrosis factor (TNF) superfamily member 15 (TNFSF15) (−2.2 fold, *P* < 7.9E-05) and miR181 (−2.4 fold, *P* < 2.1E-04) appeared specifically down-regulated in a statistical significant manner. In contrast, up-regulated transcripts in the HR vaccinated group were barely dominated by a biological function that was the response to wounding (50.2 FFE, FDR < 4.95) involving purinergic P2Y G-protein coupled receptor (P2RY12), vanin1 (VNN1) and IGF1 (Additional file 3).

### Upstream regulators using Ingenuity pathway analysis (IPA)

**Figure 6:**
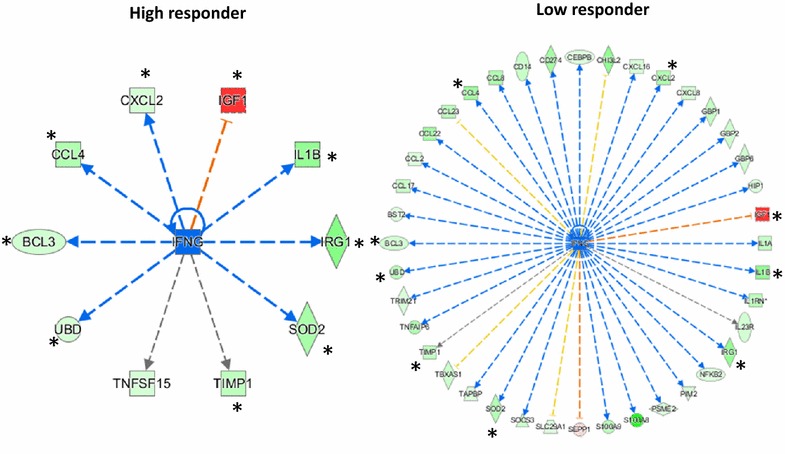
**In silico analysis of the relationship between IFNG and target genes.** Based upon the analysis of upstream regulators using IPA (see Table 3 and Additional file 4) the predicted relationship between targeted molecules and IFNG in the High responder (HR) and Low responder (LR) vaccinated groups are displayed. Ten and 40 predicted relationships were identified with IFNG and target genes in the HR and LR group, respectively. Red color denotes predicted relationships leading to activation. Blue arrows denote predicted relationship leading to inhibition. Orange color denotes inconsistencies with the state of downstream molecule. Grey color denotes effect not predicted. *Represents targeted molecules in common between LR and HR vaccinated groups.

To identify the cascade of upstream transcriptional regulators that could explain the observed gene expression data for the HR and LR vaccinated groups we performed in silico analysis using IPA. Table 3 shows top 10 upstream regulators, each predicted to act upon different sets of target molecules, likely mediating different biological effects. HR and LR datasets shared a similar set of inhibited upstream regulators (*P* < 1E-06) with the exception of IL1A and STAT3 which were specific to the LR vaccinated group (Table 3; Additional file 4). It is noteworthy that IFNG was identified as an inhibited upstream regulator with a greater activation z-score in the LR vaccinated group (−4.4, *P* value < 3.36E-20) than in the HR group (−2.4, *P* value < 1.26E-06). The results of the in silico analysis of the relationship between IFNG and target genes show that 10 and 40 predicted relationships were identified with IFNG and target genes in the HR and LR vaccinated group, respectively (Figure 6). Thus, IFNG was predicted to be an inhibited upstream regulator to explain the trend of regulation in HR and LR vaccinated groups.

**Table 3:** **Analysis of upstream regulators using IPA**

Upstream regulator	High responders		Low responders	
	Activation z-score	*P* value of overlap	Activation z-score	*P* value of overlap
TNF	−3.4	4.93E-09	−5.9	1.86E-25
Lipopolysaccharide (LPS)	−3.3	5.27E-10	−6.0	4.09E-32
IL1B	−2.8	1.81E-09	−5.2	1.08E-28
NFkB (complex)	−2.7	4.98E-08	−4.5	1.32E-15
Mma_DMAG	−2.6	2.12E-12	−3.9	6.94E-20
Immunoglobulin	−2.5	1.56E-09	n/a	n/a
IFNG	−2.4	1.28E-06	−4.4	3.36E-20
Salmonella enterica-LPS	−2.4	1.66E-08	−3.1	1.61E-12
poly rI:rC-RNA	−2.4	2.11E-06	−4.3	1.20E-16
*E. coli* B5-LPS	−2.3	1.53E-07	−4.1	1.33E-16
STAT3	n/a	n/a	−4.0	1.82E-16
IL1A	n/a	n/a	−3.9	2.49E-22

Data with *P* value < 0.05 and ¦activation Z-score¦ ≥2 were considered significant. Information related to this table can be complemented by data reported by Additional file 4 which shows target molecules of the whole datasets and associated mechanistic pathway.

Mma_DMAG, 5-*O*-mycolyl-beta-araf-(1→2)-5-*O*-mycolyl-alpha-araf-(1→1′)-glycerol [55]; n/a, not applicable (not found in the dataset). The *P* value of overlap, which indicates possible upstream regulators, represents the significance of the overlap between the dataset genes identified and the known targets of transcriptional regulators. The activation z-score was used to infer the state of activation of upstream regulators based on a comparison with a model that assigns random regulations.

## Discussion

It is increasingly recognized that the control of PRRS will require a global approach that will consider relevant factors of the pathogen, the host and the environment. Although valuable tools, current PRRSV vaccines are not fully efficacious and some vaccinated animals may still become infected [43]. It is thought that this partial lack of efficacy may be attributable to the genetic and antigenic diversity of the virus. However, several studies have highlighted that there is high inter-individual variability in the development of the immune response and protection after vaccination with a PRRSV vaccine [12, 44, 45] which may account for some lack of efficacy. Therefore one of the challenges of PRRS control is the development of tools to predict the efficacy of vaccines with greater accuracy.

To date, measurement of humoral immunity (neutralizing antibodies) and cell-mediated immunity (IFN-γ responses levels) have been used for monitoring PRRSV infection [20, 21, 46]. We hypothesized that the generation and the maintenance of protective immunity to PRRSV vaccine are a complex process that entails the regulation of transcriptional pathways leading to the production of neutralizing antibody and the induction of cell-mediated immunity. Thus, in the present study we have focused primarily in the PRRSV-specific IFN-γ responses after vaccination as a mean of discriminating between animals. The three animals with the most extreme results in the ELISPOT were selected. Our results show that in a group of cross-bred pigs of the same origin, responses to PRRSV vaccination may range from low IFN-γ responses and low or nil NA, as in the Low Responders (LR) group, to higher IFN-γ responses and moderate titers of NA, in the High Responders (HR) group. These differences indicate that inter-individual variability in markers of PRRSV vaccine-induced immunity, including neutralizing antibody levels and INF-γ responses, may be regulated, at least in part by host genetic factors [47]. This observation is indeed reminiscent with current trends suggesting that host genetic variation in the outcomes of PRRSV infections occurs in growing pigs as well as during reproduction [25]. Specifically, a QTL on chromosome SSC4 was found to be associated with viraemia levels and the growth rate in experimentally infected pigs [28]. Further, the SNPs defining this QTL have been shown to be associated with resistance and performance in several independent populations [48–50]. Collectively, these studies suggest that immune response to PRRSV is likely the results of multigenic influences and possibly not of a single dominant gene/allele and advocate the need for further genetic investigation of the host response to PRRSV vaccine.

To further delineate which genes and pathways are associated with HR and LR vaccinated pigs we have conducted a genome–wide transcriptional study of PBMCs 6 weeks post vaccination. The selection of the time-points was based on the likelihood of having the minimal influence with residual vaccine virus in blood [51] and to choose a moment in the development of the immune response when cell-mediated immunity was more or less in a steady state [11, 22].

The number of examined animals per group can represent a critical factor to reach statistical power for comparisons for such studies. Indeed, the high inter-individual variability in gene expression of control animals noticed using Biolayout Express3D software could explain why statistical significance was not achieved among vaccinated and unvaccinated groups after vaccine treatment (Table 2; Figure 4). However, the results of gene expression after vaccine stimulation showed an enrichment of immune-related genes in the LR and HR vaccinated groups as compared to the unvaccinated controls. Most of these genes, CXCL9, CXCL10 and CXCL11 were related with monocyte and T-lymphocyte activation and with interferon-modulation antiviral responses (CYP3A46, CYP3A29 and OAS1) [41, 42]. For this reason we considered that these changes in gene expression were potentially biologically relevant despite not being selected using the Anova test with Partek software. On the other hand and despite the limited number of pigs tested, statistical significance was reached for the other group comparisons (mock and PHA stimulated) suggesting that future transcriptional study of vaccine efficacy could benefit from larger number of animals per experimental group.

Gene regulation in the LR vaccinated group suggested that in those animals there was a functional impairment in at least some components of the inflammatory response such as CCL2 or IL1A but also that transcript levels or transcription of genes encoding some chemokines that act as strong chemotactic compounds for T cells, NK cell, dendritic cells and monocytes/macrophages are affected (CCL17, CCL22, CCL23). Interestingly down-regulated transcripts encoding guanylate-binding proteins GBP2, 4 and 6 were identified. Members of the GBP gene family are associated with antiviral activity in humans and as discussed previously by Boddicker et al. [28] they have been highlighted as potential candidate genes for the QTL identified on SSC4 which explains 15% of the variance in genomic estimated breeding values for viral load and 11% for weight gain 42 days post infection [48]. Recently, an intronic SNP in GBP5 has been identify as a strong candidate causal mutation for the SSC4 QTL that controls variation in host response to PRRSV [49]. As regards the HR pigs, either the lower proportion of inhibited up stream regulators of the IFN-γ cascade and the up-regulation of transcripts encoding for molecules involved in anti-apoptosis mechanisms (P2RY12, VNN1 and IGF1), suggests increased chemotactic activity, viral-induced-apoptosis resistance and a better induction of cellular immune response.

Curiously, HR vaccinated animals presented a down-regulation of a member of the tumor necrosis factor superfamily, TNFSF15, and miR181 transcripts. TNFSF15 is produced by inflammatory cytokine-stimulated endothelial cells and by TLR ligands-stimulated antigen presenting cells, and it is involved in apoptosis, cell proliferation and polarization to Th1 and Th17 responses [52]. Recently, increased levels in sera and genetic polymorphisms of TNFSF15 have been observed in inflammatory diseases that are caused by altered immunological reactions such as Crohn’s disease or ulcerative colitis [52]. As regards to miR181, over-expression of this molecule has been recently identified in minimally PRRSV-permissive cells or tissues, and this molecule is able to inhibit PRRSV replication by both mechanisms: binding to a conserved region in the downstream of open reading frame 4 [53] and downregulating the PRRSV receptor CD163 in blood monocytes and porcine alveolar macrophages [54]. The fact that TNFSF15 and miR181 were found down-regulated in HR vaccinated pigs is noteworthy and warrant further studies. It is possible that these factors may be associated with either protective mechanism induced against harmful effects of an exacerbated inflammatory response and/or a higher cell permissibility to the vaccine virus and thus, better priming and immune stimulation.

It is likely that at 6 weeks PV the transcriptional response we have measured may have been mostly associated with the resolution phase rather than the primary response. However, the finding that the transcriptional signature in LR pigs was associated with a down-regulation of the IFN-γ response was interesting for several reasons: this specific transcriptional signature was consistent with the low IFN-γ level found in the LR or the HR pigs maybe resolving some of the vaccine response. On the other hand, the results of the experiment are compatible with either a faster or a stronger response in the HR group. In each case, implications would be a little different. If the differences were related just to the intensity, the outcome of vaccination in LR could maybe be ameliorated by use of adjuvants; if the differences relied in the velocity of the immune response this could be more much difficult to solve. However, in practical terms, in situations where the risk of entering in contact with the virus are high, the point is to achieve the fastest and strongest possible response. As shown here, at 42 days PV a group of animals was far from having that type of immunity and this seemed to be related to some elements in the genetics of the pig.

In summary, this works showed that variable responses to PRRSV vaccination may be the result of the genetic characteristics of the host. If this was to be proven in future challenge studies, genetic selection of pigs could be used to increase resistance to the infection through vaccination. Taken together this work advocate the need for a greater understanding of the host genomics/genetics of PRRSV vaccine response.

## Additional files

**Additional file 1.** **List of regulated transcripts with** ***P*** **value** **<** **0.001, FDR** **<** **0.2 and fold change above 2 and below -2 in PBMCs of HR and LR pigs are displayed.** The lists of transcripts were generated using Partek software and only non-redundant records are shown HR and LR vaccinated pigs and HR and LR vaccinated pigs in presence of PHA. To enhance the comparison between the HR and LR datasets *P* value and fold change are shown across multiple experimental groups.

**Additional file 2.** **Validation of the microarray data using real time qPCR.** A subset of 9 regulated transcripts was reassessed using real-time qPCR. Data were used to run a regression analysis between the microarray and the real time qPCR experiment and generate the correlation coefficients R^2^.

**Additional file 3.** **Gene ontology analysis of the data using DAVID bioinformatics resources** [39]. Analysis was run to generate the most likely gene ontology term for the biological processes, cellular components and molecular functions for each group of regulated transcripts in HR and LR vaccinated pigs based on *P*-value, number of gene component and fold enrichment. Only data with FDR <5% are shown.

**Additional file 4.** **Upstream regulators analysis using Ingenuity Pathway Analysis for the HR and LR vaccinated pigs.** As described in material and methods section the expression dataset for HR and LR pigs were analysed independently with Fisher’s exact test. To create this dataset, activation z-score| ≥2 for HR and LR pigs were generated and ranked from the highest to the lowest. Data were considered significant with *P* value < 0.05 and ¦activation Z-score| ≥2. The activation z-score was used to infer the state of activation of upstream regulators based on a comparison with a model that assigns random regulations. Target molecules are shown for each upstream regulator.
